# Retrobulbar Sinus Injection of Doxorubicin is More Efficient Than Lateral Tail Vein Injection at Inducing Experimental Nephrotic Syndrome in Mice: A Pilot Study

**DOI:** 10.1177/0023677218824382

**Published:** 2019-01-24

**Authors:** Bernhard N. Bohnert, Thomas Dörffel, Sophie Daiminger, Carsten Calaminus, Sandro Aidone, Almuth Falkenau, Antje Semrau, Mai J. Le, Franz Iglauer, Ferruh Artunc

**Affiliations:** 1 Department of Internal Medicine, Division of Endocrinology, Diabetology, Vascular Disease, Nephrology and Clinical Chemistry, University Hospital Tübingen, Germany; 2 Institute of Diabetes Research and Metabolic Diseases (IDM) of the Helmholtz Centre Munich, Eberhard Karls University Tübingen, Germany; 3 German Centre for Diabetes Research (DZD), Eberhard Karls University Tübingen, Germany; 4 Werner Siemens Imaging Centre, Eberhard Karls University Tübingen, Germany; 5 Institute of Veterinary Pathology, Ludwig-Maximilians-University, Germany; 6 Facility for Animal Welfare, Veterinary Service and Laboratory Animal Science, Eberhard Karls University of Tübingen, Germany

**Keywords:** 3R, refinement, retrobulbar sinus injection, lateral tail vein injection, experimental nephrotic syndrome, doxorubicin, mice

## Abstract

Doxorubicin-induced nephropathy in mice is a model for studying experimental nephrotic syndrome. It corresponds to puromycin aminonucleoside nephrosis in rats. In this model, susceptible 129 S1/SvImJ mice are administered a rapid intravenous injection that can be accomplished via either the lateral tail vein or the retrobulbar sinus. Because doxorubicin is a highly toxic substance, extravasation must be avoided during the administration of the intravenous injection to prevent the development of large necrotizing lesions and exacerbation of the animals’ stress. In the present study, we compared the safety and stress of these two injection routes by using histopathological analyses of the animals’ orbital cavities or tails, respectively. The injection of 14.5 µg/g body weight doxorubicin into the mice’s lateral tail veins (*n* = 9) or retrobulbar sinuses (*n* = 19) caused no clinically detectable stress or impairment. Histopathologies of the specimens five days after doxorubicin injection revealed inflammatory lesions at the injection sites in both groups. In the orbital sinus specimens from the retrobulbar-injected group, fibrosis was evident 25 days after injection. Moreover, while all of the retrobulbar-injected mice (100%) developed nephrotic syndrome, tail vein-injected mice had a significantly lower response rate (66%, *p* = 0.047, Fisher’s exact test) and exhibited only attenuated features of nephrotic syndrome. It was therefore concluded that doxorubicin administration via either lateral tail vein or retrobulbar sinus injections led to a similar induction of histopathological changes with no effects on the clinical well-being of the mice. However, retrobulbar sinus injections were more efficient for inducing experimental nephrotic syndrome.

## Résumé

La néphropathie induite par la doxorubicine chez la souris est un modèle utilisé pour étudier le syndrome néphrotique expérimental. Il correspond à la néphrose par puromycine aminonucléoside chez le rat. Dans ce modèle, des souris 129 S1/SvImJ susceptibles ont reçu une injection intraveineuse rapide qui peut être administrée dans la veine caudale latérale ou le sinus rétro-oculaire. Parce que la doxorubicine est une substance hautement toxique, l'extravasation plasmatique doit être évitée pendant l'administration de l'injection intraveineuse afin d'empêcher le développement de grandes lésions nécrosantes et l'exacerbation du stress des animaux. Dans la présente étude, nous avons comparé la sécurité et le stress de ces deux voies d'injection en utilisant des analyses histopathologiques des cavités orbitales ou de la queue des animaux, respectivement. L'injection de 14,5 µg/g de poids corporel de doxorubicine dans les veines de la queue latérale de la souris (n = 9) ou les sinus rétrobulbaires (n = 19) n'a causé aucun stress ni aucune déficience cliniquement décelable. Les histopathologies des spécimens cinq jours après l'injection de la doxorubicine ont révélé des lésions inflammatoires au point d'injection dans les deux groupes. Dans les spécimens de sinus orbital du groupe recevant une injection rétro-oculaire, une fibrose était évidente 25 jours après l'injection. En outre, alors que l'ensemble des souris ayant reçu une injection rétrobulbaire (100%) a développé un syndrome néphrotique, celles qui ont reçu une injection dans la veine de la queue avaient un taux de réponse sensiblement moins élevé (66%, p = 0,047, test exact de Fisher) et ne présentaient que des caractéristiques atténuées du syndrome néphrotique. Il a donc été conclu que l'administration de doxorubicine par injection dans la veine caudale latérale ou le sinus rétrobulbaire conduisait à une induction de changements histopathologiques similaires et n'avait aucun effet sur le bien-être clinique de la souris. Les injections dans le sinus rétrobulbaire étaient cependant plus efficaces pour induire un syndrome néphrotique expérimental.

## Abstract (de)

Die Doxorubicin induzierte Nephropathie bei Mäusen ist ein Modell zur Untersuchung des experimentellen nephrotischen Syndroms. Es entspricht der Puromycin-Aminonukleosid-Nephrose bei Ratten. In diesem Modell wird empfänglichen 129 S1/SvImJ-Mäusen eine schnelle intravenöse Injektion verabreicht, die entweder über die laterale Schwanzvene oder den retrobulbären Sinus durchgeführt werden kann. Da Doxorubicin eine hochgiftige Substanz ist, muss bei der Verabreichung eine ein Paravasat vermieden werden, um die Entstehung großer nekrotisierender Läsionen und eine zusätzliche Belastung für die Tiere zu verhindern. In der vorliegenden Studie haben wir die Sicherheit und die Belastung dieser beiden Injektionswege anhand histopathologischer Analysen der Orbitalhöhlen bzw. Schwänze der Tiere verglichen. Die Injektion von 14,5 µg/g Körpergewicht Doxorubicin in die seitliche Schwanzvene (n = 9) oder den retrobulbären Sinus (n = 19) der Mäuse verursachte keine klinisch erkennbare Belastung oder Beeinträchtigung. Die Histopathologie der Proben fünf Tage nach der Doxorubicin-Injektion zeigte in beiden Gruppen entzündliche Läsionen an den Injektionsstellen. In den Proben der retrobulbären Injektionsgruppe war 25 Tage nach der Injektion eine Fibrose erkennbar. Während alle retrobulbär injizierten Mäuse (100 %) ein nephrotisches Syndrom entwickelten, hatten Mäuse mit Schwanzveneninjektion eine deutlich niedrigere Ansprechrate (66 %, p = 0,047, Exakter Test nach Fisher) und zeigten nur abgeschwächte Merkmale des nephrotischen Syndroms. Zusammenfassend lässt sich festhalten, dass die Verabreichung von Doxorubicin über die laterale Schwanzvene oder die retrobulbäre Sinusinjektion zu einer ähnlichen Induktion histopathologischer Veränderungen ohne Auswirkungen auf das klinische Wohlbefinden der Mäuse führte. Im Hinblick auf die Induktion des nephrotischen Syndroms war jedoch die retrobulbäre Sinusinjektion effizienter.

## Resumen

La nefropatía inducida con doxorrubicina en ratones en un modelo para estudiar el síndrome nefrótico experimental. Corresponde a la nefrosis aminonucleósida puromicina en ratas. En este modelo, los ratones 129 S1/SvImJ susceptibles son administrados una inyección intravenosa rápida que puede completarse mediante la vena de la cola lateral o el sinus retrobulbar. Ya que la doxorrubicina es una sustancia altamente tóxica, debe evitarse la extravasación durante la administración de la inyección intravenosa para evitar el desarrollo de lesiones necrotizantes grandes y la exacerbación del estrés de los animales. En el estudio actual, hemos comparado la seguridad y el estrés de estas dos rutas de inyecciones utilizando análisis histopatológicos de las cavidades orbitales o las colas de los animales, respectivamente. La inyección de doxorrubicina en un peso corporal de 14.5 µg/g de las venas de la cola lateral de los ratones (n = 9) o sinusis retrobulbares (n = 19) no provocaron ningún estrés o impedimento clínicamente detectable. Las histopatologías de los especímenes cinco días después de la inyección de doxorrubicina revelaron lesiones inflamatorias en los lugares de la inyección en ambos grupos. En los especímenes de sinus orbital del grupo inyectado retrobulbarmente, se detectó fibrosis 25 días después de la inyección. Asimismo, mientras todos los ratones inyectados retrobulbarmente (100%) desarrollaron el síndrome nefrótico, los ratones inyectados por la vena de la cola obtuvieron un porcentaje de respuesta mucho más inferior (66%, p = 0.047, test exacto de Fisher) y mostraron únicamente síntomas leves del síndrome nefrótico. Por tanto se pudo concluir que la administración de doxorrubicina a través de inyecciones en la vena de la cola lateral o el sinus retrobulbar llevaron a una inducción similar de los cambios histopatológicos sin efectos en el bienestar clínico de los ratones. Sin embargo, las inyecciones sinus retrobulbares fueron más eficientes para inducir el síndrome nefrótico experimental.

## Introduction

Doxorubicin-induced nephropathy as a model for inducing experimental nephrotic syndrome in mice was first published in 2008.^1^ It corresponds to puromycin aminonucleoside nephrosis in rats^2^ and uses genetically susceptible 129 S1/SvImJ mice and rapid intravenous injection into the retrobulbar sinus.^1,3,4^ The resulting nephrotic syndrome exhibits numerous features of proteinuric kidney disease. These range from non-nephrotic proteinuria to massive nephrotic syndrome with proteolytic activation of the epithelial sodium channel (ENaC), sodium retention, oedema formation and endocrinological dysregulations, such as secondary hyperparathyroidism and renal anaemia.^1,3–7^ Histologically, this model is characterized by non-inflammatory podocyte loss resulting in glomerulosclerosis, secondary interstitial fibrosis and tubular atrophy.^1,3^ Compared to other models, such as 5/6 nephrectomy,^8,9^ unilateral ureter ligation^10^ and genetic models,^11,12^ currently utilized in nephrology research, doxorubicin-induced nephropathy most closely exhibits the features of human chronic kidney disease (CKD) and nephrotic syndrome. In particular, it is the only model to show oedema formation with significant sodium retention;^1,3–6^ thus, it is the best model for investigating highly topical scientific questions such as proteasuria.^4–6,13^ Additional advantages of this model include the low cost of doxorubicin, the lower complexity facilitating management, good reproducibility^14^ and the ability of doxorubicin to induce renal injury after a single dose.^1–6,15–17^

Despite its advantages, this model has been used by relatively few researchers.^1,3–6,18,19^ A reason could be the demanding retrobulbar sinus application route for doxorubicin, a highly tissue-toxic drug.^4,20–23^ A second difficulty is the importance of the total bioavailability of the injected doxorubicin for the induction of nephrotic syndrome. Differences of just 0.5 µg/g body weight (bw) in the injected dose can cause resistance to model induction, especially in mice.^24,25^

Retrobulbar and tail vein injections are two widely published intravenous approaches for mice. Several studies have shown that retrobulbar and tail vein injections are equally safe and effective.^26–28^ However, use of the retrobulbar injection site for highly toxic substances such as doxorubicin is frequently discouraged^26,29^ because of concerns about the potential damage to the eye and vision resulting from the blind injection, which could cause undetected extravasation and increase stress.^29–34^ Institutional animal care and use committees (IACUCs), particularly those in Germany but also elsewhere in Europe, have therefore recommended restrictions for the use of this method. Specifically, they recommend that only highly trained persons be allowed to use this technique and that irritant and toxic substances be avoided.^29^ The purpose of the present study was therefore to compare lateral tail vein injections and retrobulbar sinus injections in terms of both animal safety and the effective induction of experimental nephrotic syndrome.

## Methods

### Animals

Experiments were performed on 3-month-old wild-type 129 S1/SvImJ mice of both sexes purchased from Jax Mice, USA. The mice were kept under specific-pathogen-free (SPF) conditions (Table 1) with a 12:12-h light-dark cycle at 22 ± 2℃ and 45–65% humidity. They were fed a standard diet (ssniff, Soest, Germany) with tap water ad libitum (Stadtwerke Tübingen, Germany; Na^+^ 8 mg/l, K^+^ 1.7 mg/l).^35^ They were housed in Type II cages (Tecniplast, Hohenpeißenberg, Germany) with aspen wood chips as bedding (Aspen Animal Bedding AB6, AsBe-wood GmbH, Buxtehude, Germany) and red polycarbonate mouse houses and cellulose paper for nesting material and enrichment.^36^ To more accurately monitor the animal food and drink intake after the doxorubicin injection and during nephrotic syndrome, the mice were housed individually in visual, auditory and olfactory contact with other mice.

**Table 1.** Specific-pathogen-free (SPF) status. SPF conditions excluded the following microorganisms.

Viruses	Bacteria	Fungi	Parasites
Kilham rat virus	*Bordetella bronchiseptica*	Trichophyton	*Aspiculuris tetraptera*
Ectromelia virus	*Clostridium piliforme* (Tyzzer disease)	*Microsporum canis*	*Syphacia muris*
Sendai virus	*Corynebacterium kutscheri*		*Syphacia obvelata*
Sialodacryoadenitis/ rat coronavirus	*Leptospira*		*Trichossomoides crassicaule*
Hantavirus	*Mycoplasma* spp.		*Strongyloides ratti*
Mouse adenovirus	*M. pulmonis*,		*Capillaria hepatica*
Reovirus type 3	*M. arthritidis*		*Multiceps serialis* larva
Mouse hepatitis virus	*Salmonella* spp.		*Hydatigera taeniaeformis* larva
Theilevirus	*Streptobacillus moniliformis*		*Hymenolepis* sp.
Rotavirus			*Leptopsylla segnis*
Lymphocytic choriomeningitis virus (LCMV)			*Myobia musculeux*
Minute virus of mice (MVM/MPV)			*Myocoptes musculosa*
			*Nosopsyllus fasciatus*
			*Notoedres muris*
			*Sarcoptes scabiei*
			*Polyplax* sp*.*
			*Psoregates simplex*
			*Radfordia* sp*.*
			*Trichoecius* sp*.*
			*Hoplopleura* sp.

### Study design

This pilot study had a small sample size, as specified by the local IACUC. The aim was to compare the established procedures for retrobulbar injections with those for lateral tail vein injections, which are considered safer by the German Society of Laboratory Animal Science (GV-SOLAS) whose recommendations are the basis for German IACUC specifications. Experimental nephrotic syndrome was induced after a single intravenous injection of doxorubicin (14.5 µg/g bw, 2.0 µg/µl doxorubicin; Cell Pharm, Bad Vilbel, Germany). Physiological saline solution (0.9% NaCl, 7.25 µl/g bw; Fresenius Kabi Deutschland, Bad Homburg, Germany) was injected into the control mice.

For the retrobulbar sinus injections, 15 mice were divided into three groups. Five mice were treated with doxorubicin and euthanized on Day 5 after injection, five were treated with NaCl and euthanized on Day 5 after injection, and five were treated with doxorubicin and euthanized on Day 25 after injection. Euthanasia for all the animals was achieved by anaesthesia with isoflurane followed by decapitation. Subsequently, the skin was removed from the head, which was placed in 4.5% formaldehyde (SAV Liquid Production GmbH, Flintsbach am Inn, Germany).

Because nephrotic syndrome develops after five days, the mice euthanized on Day 5 could not be used for assessing the response rates. The response rates were assessed for the group euthanized on Day 25 (*n* = 5) and a historic cohort of mice (*n* = 9). This cohort from a previous study^5^ had undergone the same treatment. Thus, the yield was a total of 14 mice that had received retrobulbar injections of doxorubicin.

In addition, 10 mice received lateral tail vein injections. Of these, nine were treated with doxorubicin, and one, with saline. All of these mice were euthanized on Day 10 after which sharp shears were used to cut off the tails at the proximal end. The tails were subsequently stored in 4.5% formaldehyde. The response rates for the mice that had received the tail vein injections of doxorubicin (*n* = 9) was then compared to those for the mice that had received the retrobulbar injections (*n* = 14).

### Injection techniques

For the lateral tail vein injections, an improvised catheter was used, which consisted of the tip of a 30 G insulin-injection cannula (Sterican® Insulin G 30 × 1/2''/Ø 0.30 × 12 mm, B. Braun Melsungen AG, Melsungen, Germany), a tube approximately 10 cm long (THOMAFLUID® High-Tech LDPE tube, inner diameter 0.28 mm, outer diameter 0.61 mm, Reichelt Chemietechnik GmbH + Co., Heidelberg, Germany) and a 0.5 ml insulin syringe (BD Micro FINE™ + U-40, 0.30 mm × 8 mm, BD Deutschland GmbH, Heidelberg, Germany). Before the catheter was placed into the lateral tail vein, the mice were lightly anaesthetized, as described above. Thereafter, dilatation of the tail veins was induced by local heating in a 45℃ water bath for 30 s. After drying, the catheter was inserted into the distal tail. The correct intravascular position was ascertained by the backflow of blood and the administration of a 50 µl bolus of rinsing solution (500 IE heparin/10 ml 0.9% saline) without any resistance. If this was unsuccessful, catheter insertion was then reattempted in a more proximal position. To empty the catheter of doxorubicin and to ensure the administration of a full dose, an additional 25 µl bolus of rinsing solution was given after the doxorubicin injection. During the entire procedure, a funnel-shaped nose cone was used to administer a dose of 1.5 vol% isoflurane to the mouse. To avoid hypothermia, the mice were placed on a warming device (Thermo MAT Pro 10 W, 15 × 25 cm, Lucky Reptile, Import Export Peter Hoch GmbH, Waldkirch, Germany) covered with a layer of gauze to avoid thermal injuries.

### Monitoring after injections

Samples of spontaneously voided urine were collected in the morning (8.00 a.m.) before doxorubicin injection, i.e. at baseline, and from Days 5–10 following injection. Food and fluid intake, as well as animal well-being, were monitored daily. Table 2 presents the animal well-being scoresheet, which is in accordance with Morton and Griffiths^37–40^ and Langford et al.^41^ Table 3 shows the scoresheet that was used for monitoring adverse doxorubicin injection effects, such as signs of neurotoxicity or injury at the injection sites.

**Table 2.** Daily scoresheet for assessment of well-being. The maximum reachable score was 20. At a score of 7 or higher, or when certain conditions were met, the mouse concerned was euthanized. From a score of 5, the mouse concerned was observed more closely: twice daily.

Parameter	Change	Score
Body weight	No change	0
	Weight loss 5–10% within 3 consecutive days	1
	Weight loss > 10–20% within 3 consecutive days	2
	Weight loss > 20% within 3 consecutive days	3 (immediate termination of experiment and euthanasia)
Drinking	No change	0
	Reduction of drinking by 5–10% within 3 consecutive days	1
	Reduction of drinking by > 10–20% within 3 consecutive days	2
	Reduction of drinking by > 20% within 3 consecutive days	3
Food intake	No change	0
	Reduction of food intake by 5–10% within 3 consecutive days	1
	Reduction of food intake by > 10–20% within 3 consecutive days	2
	Reduction of food intake by > 20% within 3 consecutive days	3
Locomotion	Normal	0
	Slowed	1
	Apathetic	2
	Moribund	3 (immediate termination of experiment and euthanasia)
Coat	Normal, glossy	0
	Shaggy/piloerection	1
	Polluted with faeces, diarrhoea	2
Breathing	Normal	0
	Hypoventilation	1
	Abdominal breathing	2
Posture	Normal	0
	Slightly bent	1
	Markedly bent	2
Mouse grimace scale (MGS) score, calculated as described by Langford et al.^35^	MGS score = 0	0
	MGS score > 0	1
	MGS score > 1	2

**Table 3.** Daily scoresheet for assessment of neurotoxicity or signs of injury at the doxorubicin injection site. The maximum reachable score was 7. At a score of 1 or higher, the mouse concerned was euthanized. A score of 0 for any given parameter indicates ‘no’, and a score of 1 indicates ‘yes’.

Parameter	Score	In case of
Mouse grimace scale	≤1 (score 0)/ >1 (score 1)	
Shaking	0/1	
Head tilt	0/1	
Manage movements	0/1	
Exophthalmus	0/1	Retrobulbar sinus injection
Enophthalmus	0/1	Retrobulbar sinus injection
Eyelid closure	0/1	Retrobulbar sinus injection
Increase in tail size	0/1	Lateral tail vein injection
Skin lesions	0/1	Lateral tail vein injection
Reduced tail movement	0/1	Lateral tail vein injection

### Laboratory assays

The urinary creatinine concentration was measured with a colorimetric assay (Labor + Technik, Berlin, Germany). The urinary protein concentration was quantified through the Bradford method (Bio-Rad Laboratories, München, Germany), and the urinary sodium concentration was quantified by flame photometry (Eppendorf EFUX 5057, Hamburg, Germany). Both the urinary protein and sodium concentrations were normalized to the urinary creatinine concentration.

### Histopathological examinations

The formaldehyde-fixed specimens were shipped to the Institute of Veterinary Pathology (LMU München) for further histomorphological examination. To facilitate later orientation under the microscope, a surgical blade was used to mark each head (*n* = 15) and tail (*n* = 10) with a left dorsolateral incision in a parasagittal orientation. The marked specimens were immersed in a slow-acting decalcification solution (Q Path, DC1, VWR BDH Chemicals). The heads were decalcified for 24 h, and the tails for 48 h.

To generate three sections of the eye and surrounding connective and supporting tissues, the decalcified heads were transversely cut four times in approximately 2 mm thick slices. Beginning at the medial canthus, cross-sections were performed through the eyes, the temporal canthus, and 2 mm caudal from the temporal canthus. The tails were transversely sectioned at 2 mm intervals, for a total of 36 sections per tail. All of the sections were routinely processed in alcohol and xylene and embedded in paraffin. Histological sections with a thickness of 3–4 µm were stained with haematoxylin and eosin (HE). Each histopathological examination was performed by an examiner without knowledge of the type of injection solution used. The intraorbital and adjacent extraorbital structures, as well as the lateral tail veins and associated connective and supporting tissues, were particularly examined for lesions. The quality and severity were documented with a semiquantitative scoring system: no lesion (–), mild lesion (+), moderate lesion (++), or severe lesion (+++).

### Statistical analysis

The data are provided as arithmetic means ± SEM, with *n* representing the number of independent experiments. The data were tested for normality with the Kolmogorov–Smirnov test, the D’Agostino–Pearson omnibus normality test and the Shapiro–Wilk test. To test the statistical significance of the response rate independence from the injection route, Fisher’s exact test was performed. The variances were tested through Bartlett’s test for equal variances. The data were tested for significance with the unpaired Student’s *t*-test or the Mann–Whitney *U*-test, as applicable. GraphPad Prism 6 software (GraphPad, San Diego, CA, www.graphpad.com) was used. A *p* value of < 0.05 with two-tailed testing was considered statistically significant.

### Study approval

All of the animal experiments were conducted according to both the National Institutes of Health’s Guide for the Care and Use of Laboratory Animals and German law for the welfare of animals, with approval from the local authorities (Regierungspräsidium Tübingen, M11/15).

## Results

### Mouse well-being after injection

**Figure 1.**
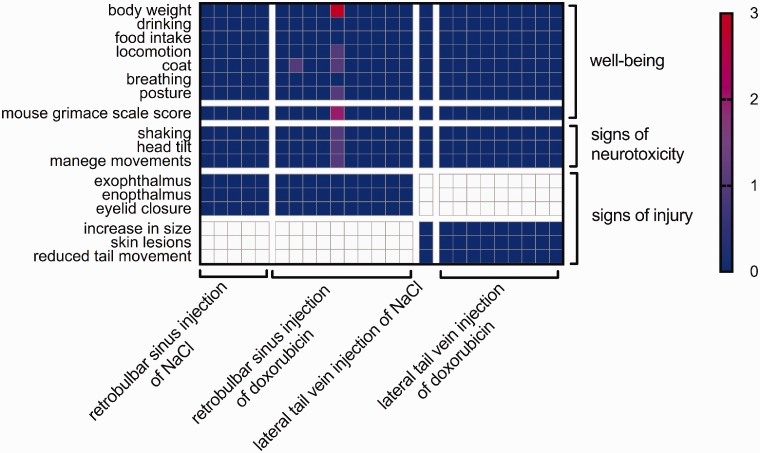
Mouse well-being after injection of saline or doxorubicin. Each column represents one mouse. The mouse values are colour-coded. For well-being, 0 (blue) was the best possible result and 3 (red) was the worst. For neurotoxicity and injury, only scores of 0 (no symptoms) or 1 (symptom detectable) were possible. Each value coded on the map is the highest value reached by the given mouse during observation. The left axis shows the categories from the well-being scoresheet that was used for daily evaluation.

For all the animals, the injection procedures were ultimately successful and without complications. While each retrobulbar injection required only one attempt, each tail vein injection in the brown-tailed 129 S1/SvImJ mice required up to five attempts for inserting the catheter safely into the vein. None of the animals developed any extravasate or macroscopically suspicious lesions at any of the injection sites during the experiment. None of the mice exhibited any of the parameters monitored by the well-being scoresheet. An exception was one mouse that had to be euthanized on Day 3 after the retrobulbar doxorubicin injection because of neurotoxicity symptoms: manege movements, head tilt and shaking (Figure 1).

### Development of nephrotic syndrome

**Figure 2.**
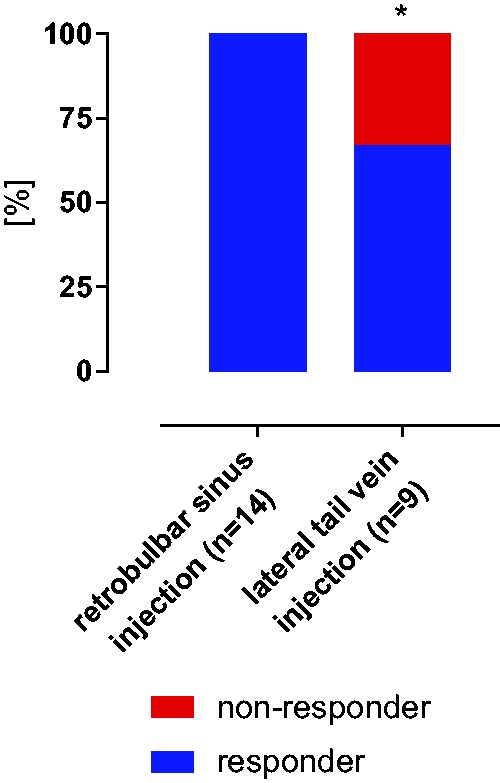
Response rate for induction of experimental nephrotic syndrome. All of the animals receiving doxorubicin via retrobulbar sinus injection developed nephrotic syndrome (proteinuria >140 mg/mg creatinine) within 10 days; however, this was true for only six of the nine mice injected via the lateral tail vein. This result corresponded to a non-response rate (red) of 33% (*p* = 0.047, Fisher’s exact test). (*indicates a significant difference between retrobulbar sinus injection and lateral tail vein injection.)

Six of the nine mice that received doxorubicin via lateral tail vein injection developed proteinuria (>140 mg/mg creatinine), a requirement for inducing nephrotic syndrome. This corresponds to a response rate of 66.6%. When doxorubicin was injected by the retrobulbar sinus route, all of the mice from this study (*n* = 5) and the previous study^5^ (*n* = 9) became nephrotic. This corresponded to a response rate of 100% (*p* = 0.047, Fisher’s exact test; Figure 2).

**Figure 3.**
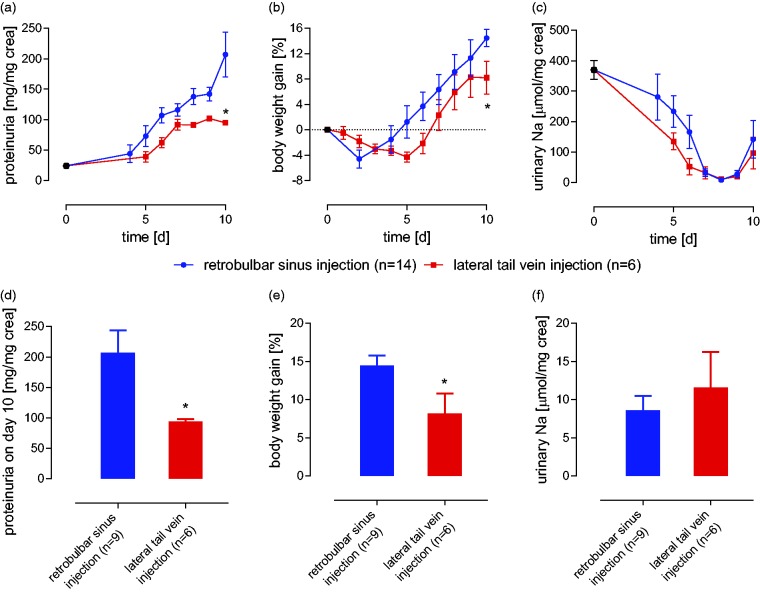
Course of experimental nephrotic syndrome after doxorubicin injections via tail vein v. retrobulbar sinus injections in mice exhibiting a response. Following retrobulbar sinus injections of doxorubicin, all the hallmarks of human nephrotic syndrome were present. This included (a) proteinuria, (b) body weight gain, and (c) urinary sodium retention. Following the lateral tail vein injections, the maximal proteinuria (D) and body weight gain (E) were less pronounced, and the minimal sodium excretion (F) was higher. (* indicates a significant difference between retrobulbar sinus injection and lateral tail vein injection (based on Student’s *t*-test and the Mann-Whitney *U*-test, as appropriate.)

In the six tail vein-injected mice that developed proteinuria, the course of nephrotic syndrome was attenuated. They exhibited significantly lower proteinuria, weight gain and higher natriuresis (Figure 3).

### Histopathological results after retrobulbar sinus injections

**Figure 4.**
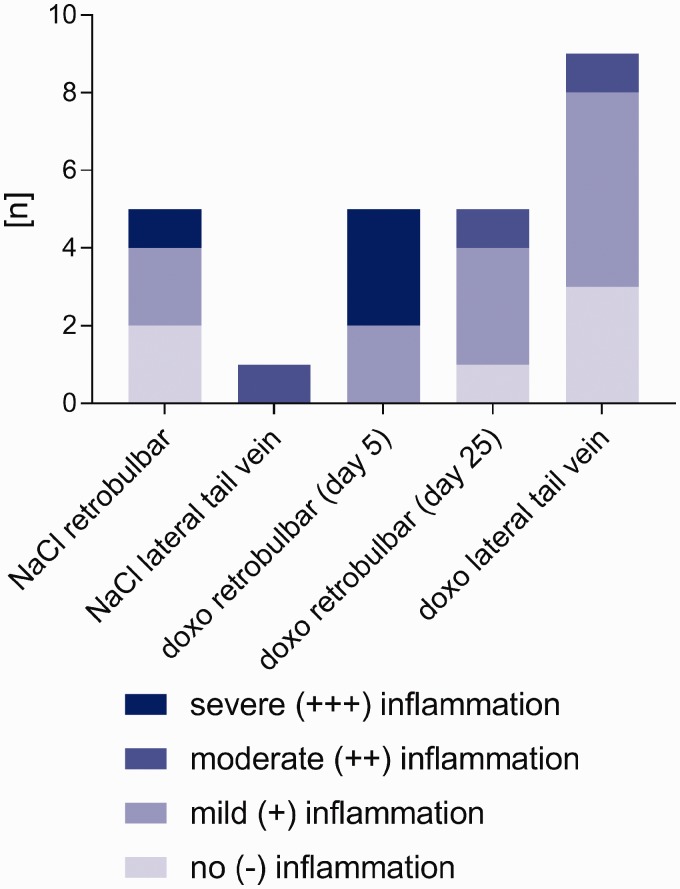
Histological results after injection of NaCl or doxorubicin via tail vein or retrobulbar sinus. Histological analyses showed local inflammation after doxorubicin (doxo) injection and after NaCl injection. This was observed after administration of the retrobulbar sinus injections and the lateral tail vein injections. The degree of inflammation ranged from no inflammation (light blue) to severe (+++) inflammation (dark blue).

**Figure 5.**
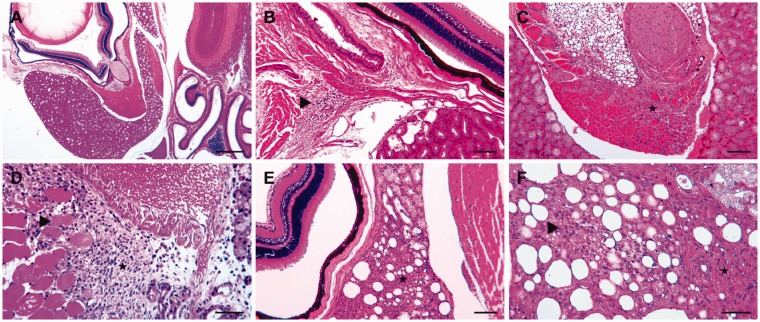
Representative sections of retrobulbar sinus-injected mice. The histological sections were stained with hematoxylin and eosin. (a) An overview of the intraorbital tissues, including the bulbus and the Harderian gland. For four of the five mice, no pathological changes were observed five days after the injection of a physiological saline solution. (b), (c) and (d) Lesions of varied severity five days after the injection of doxorubicin. (b) Focal mild inflammatory infiltration of lymphocytes and plasma cells in the retrobulbar connective tissue (▸). (c) The replacement of multiple degenerated orbital muscle fibres from severe proliferation of the granulation tissue (★). (d) The severe proliferation of granulation tissue and cell infiltration with neutrophilic granulocytes, macrophages, lymphocytes and plasma cells in the temporal muscle (★) adjacent to the orbital bone and retrobulbar sinus. Within the granulation tissue are several separated degenerated myofibers (▸). (e), (f) Examples of commonly detected lesions in mice 25 days after doxorubicin injection: (e) The medial part of the Harderian gland shows marked interstitial fibrosis and loss of multiple acini (★) between the bulbus (left) and orbital bone (right); (f) a closer view of the changes, besides fibrosis, in the Harderian gland: with prominent extracellular collagenous matrix (★) and loss of acini, as well as mild acinar regeneration (▸). Scale bars: (a) 500 µm; (b), (c), (e) 100 µm; (d), (f) 50 µm.

Of the five mice that received retrobulbar sinus injections of NaCl, two exhibited no abnormalities (–) in their intraorbital structures (Figures 4 and 5), including the eyes and Harderian glands, and the adjacent extraorbital tissues. One mouse from this group showed severe (+++) inflammation of the orbital muscles and retrobulbar connective tissues. This inflammation was comprised of cellular infiltration of several neutrophilic granulocytes, macrophages, plasma cells and a few lymphocytes. In addition, there was a proliferation of granulation tissue, with the granulation tissue replacing multiple necrotic myofibers. In addition, this mouse, along with two others in this group, displayed a unilateral mild (+) conjunctivitis of the left eye.

Mild (+) to severe (+++) inflammation of retrobulbar tissue was seen in all five mice that received retrobulbar doxorubicin injections (Figures 4 and 5). Specifically, three of the mice showed severe orbital muscle alterations with myofiber degeneration and regeneration, proliferation of granulation tissue and inflammatory cell infiltrations, which consisted of neutrophilic granulocytes, macrophages, lymphocytes and plasma cells (Figure 5(c)). Two of these mice also displayed severe (+++) lesions in the temporal muscle adjacent to the orbital bone and similar features in the orbital muscles (Figure 5(d)). These mice also showed unilateral mild (+) conjunctivitis of the left eye.

Of the five mice that were euthanized 25 days after the retrobulbar administration of doxorubicin, only one had no lesions (–) in its orbital structures. The other four mice exhibited mild (+) to moderate (++) changes in their Harderian glands (Figure 5(e) to (f)). Each of these four mice also displayed varying degrees of acinar degeneration and regeneration, interstitial fibrosis and infiltration with predominant neutrophilic granulocytes, as well as a few macrophages. Overall, the histopathological changes were less marked in these mice (Figures 4 and 5).

Of the five mice that received a retrobulbar doxorubicin injection, the incidence of inflammatory changes after five days was 100%. Of the five mice that received a retrobulbar NaCl injection, three (60%) exhibited inflammatory changes. However, a statistical analysis of this ratio with Fisher’s exact test showed the difference to be non-significant (*p* = 0.4444).

### Histopathological results after lateral tail vein injection

**Figure 6.**
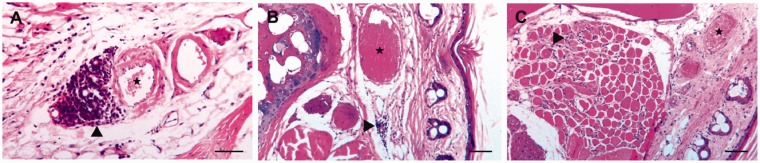
Representative sections of lateral tail vein-injected mice 10 days after injection. The histological sections were stained with hematoxylin and eosin. (a) Moderate perivascular cellular infiltration (▴) with predominant lymphocytes and fewer neutrophilic granulocytes and plasma cells after injection of NaCl into the lateral tail vein (★). (b) Milder inflammatory infiltration (▸) in the connective tissue near the lateral tail vein (★) of a mouse after injection with doxorubicin. (c) Skeletal muscle bundles in proximity to the lateral tail vein (★) with a multifocal mild increase of endomysial connective tissue, as well as mild endomysial inflammatory infiltration and loss of multiple myofibers (▸) 10 days after doxorubicin injection. Scale bars: (a) 50 µm; (b), (c) 100 µm.

Because of multiple injection attempts, seven of the 10 mice that received lateral tail vein injections showed mild to moderate inflammatory alterations at up to four sites 10 days after injection (Figure 4). There were no pathological findings in the rated slices of the other three mice. Specifically, one of the seven mice that exhibited inflammation had mild (+) focal perivascular lymphoplasmacellular inflammation of the lateral tail vein and mild (+) diffuse endomysial fibrosis of the entire skeletal muscle in the same localization. Another mouse exhibited a similar perivascular lesion, but the mild (+) fibrosis and lymphocytic infiltration of the endomysium were limited to the skeletal muscles in close proximity to the altered lateral tail vein. Examinations of the tails of the other five mice revealed similar mild (+) lesions on the skeletal muscles adjacent to the unaltered lateral tail vein. One of these mice also exhibited multiple myofiber degenerations. Furthermore, two mice showed mild (+) lymphocytic and neutrophilic infiltration of the connective tissues in proximity to the lateral tail vein. One of these mice also displayed mild (+) inflammatory infiltrations of the subcutaneous connective tissues at two other sites. Another mouse displayed only mild (+) and predominant lymphocytic infiltration of subcutaneous stroma at two sites without distinct proximity to the lateral tail vein. One mouse tail showed an inflammatory response to a displaced keratin disc in the subcutaneous connective tissue. The mouse treated with NaCl also exhibited perivascular inflammation of the lateral tail vein with infiltration of the neutrophilic granulocytes, lymphocytes and a few plasma cells at two different sites to a moderate (++) extent (Figures 4 and 6).

## Discussion

The results indicate that doxorubicin administered via retrobulbar sinus injection was more reliable than doxorubicin administered via lateral tail vein injection for inducing experimental nephrotic syndrome in mice. Although doxorubicin is a highly toxic substance, the histopathologic lesions observed after the retrobulbar injections were comparable to those observed after the lateral tail vein injections. Most important, the doxorubicin had no effect on the overall clinical evaluation of the mice. The mice that received the lateral tail vein injections exhibited a significantly higher proportion of non-response (33%) than those that received the retrobulbar sinus injections (0%). This was roughly consistent with the results of a previous study with a much larger group of animals (*n* = 128). That study reported a non-response in 15% of animals after retrobulbar sinus injection (*p* = 0.1579, Fisher’s exact test).^5^ Further data from a larger cohort of tail vein-injected mice are unfortunately not available; thus, this is a limitation of the study.

Moreover, the six mice that developed proteinuria after receiving lateral tail vein injections showed attenuated symptoms of nephrotic syndrome. In this mouse model, sodium retention and body weight gain depend on the extent of ENaC activation by proteinuria or proteasuria.^4–6^ Therefore, the lower proteinuria exhibited in the tail vein-injected mice suggests less podocyte injury from the doxorubicin.^1,2^

Given that the doxorubicin dose was the same regardless of the administration method, the differences in response rates would be explained by pharmacokinetics. It is conceivable that tail vein injections lead to a lower peak concentration in the plasma, which plays a central role in model induction.^24^ This lower peak plasma concentration might be explained by the higher overall injection volume resulting from the use of a flushing solution in the tail vein-injected mice. This was necessitated by the use of a catheter. In addition to getting the already high volume of doxorubicin solution (7.25 µl/g bw), these mice received 75 µl (or *c.* 3 µl/g bw) fluid. This would inevitably have led to significantly higher plasma volume expansion and dilution. In a similar study comparing the distribution kinetics of contrast media in mice, Socher et al*.* showed that tail vein-injected contrast media were dissolved below the diaphragm and were only slowly transported to the heart. In contrast, an injection into the retrobulbar sinus was followed by a rapid transport to the heart and arterial system.^42^ This altered distribution was also likely true for the doxorubicin route to the kidneys. A dose increase might have compensated for the reduced response rates with tail vein injection; however, the dose of doxorubicin used for model induction by retrobulbar injection (14.5 µg/g bw) was already close to the described median lethal dose (LD 50) of 15–17 µg/g body weight^1,43^ in this mouse strain.

Histopathological analyses showed local inflammatory reactions after both doxorubicin and NaCl injections at the retrobulbar sinus and lateral tail vein injection sites. However, the degree of inflammatory reaction following the doxorubicin injections was significantly higher, as would be expected for a highly toxic and irritant substance. The absence of large necrotizing lesions in the mice that received tail vein and retrobulbar sinus injections were evidence of the complete intravenous application of the entire volume of doxorubicin. The smaller degenerative and necrotizing lesions observed in this study have also been previously described by other groups after injections of non-toxic substances. They probably originated from the tissue damage caused by the injection canula.^44^ It must be underscored that the histopathological findings, although more pronounced after doxorubicin treatment, had no effect on the macroscopic integrity of the injection sites or the overall clinical evaluations of the mice.

When stress and harm to the animals is considered, the induction of nephrotic syndrome via tail vein injection is by no means less intense. On the contrary, the higher non-responder rate would lead to an overall higher burden because of the larger group size. Moreover, the attenuated responses, i.e. lower proteinuria, lower body weight gain and higher natriuresis, would complicate the detection of effects between different treatment groups or genotypes. The number of animals would therefore have to be further increased to achieve sufficient statistical power. This would violate the 3 R principle of reduction.^45^

The observance of the overall recommendations of various IACUCs^29^ that retrobulbar sinus injections be performed only if absolutely necessary and only by highly trained operators is crucial. However, under these conditions and with highly experienced operators,^1,3–6^ retrobulbar sinus injection, even with a highly toxic substance like doxorubicin, is an efficient and reliable alternative to intravascular access. This method of injection has been legitimized by its different injection kinetics. This is true especially for experiments, such as the determination of glomerular filtration rates (GFR) ^46,47^ and the induction of podocyte damage by doxorubicin,^3,24^ that are dependent upon fast and high plasma peak concentrations. Beyond nephrology, there are also other uses, such as contrast media applications in radiological imaging studies, that are critically dependent upon rapid intravenous injection.^42^

## Conclusion

The results of this study indicate that doxorubicin-induced nephropathy on the basis of retrobulbar sinus injection is an efficient and reliable method for inducing nephrotic syndrome in mice. Lateral tail vein injections did not reduce animal stress. They led to a higher non-responder rate in this model and also attenuated the features of nephrotic syndrome in the responder mice. The histological lesions indicate that the injections at both the retrobulbar sinus and the lateral tail vein were not harmless. In summary, doxorubicin-induced nephropathy via retrobulbar sinus injection remains the only mouse model known to facilitate the study of experimental nephrotic syndrome with sodium retention.
